# What happens after the kidney biopsy? The findings nephrologists should know

**DOI:** 10.1186/s12882-022-02881-w

**Published:** 2022-07-25

**Authors:** Daniel Montes, Claire Beamish, Sana Waheed, Fauzia Osman, Laura Maursetter

**Affiliations:** 1 School of Medicine and Public Health, University of Wisconsin, 600 Highland Ave, Madison, WI 53705 USA; 2 Piedmont Nephrology and Internal Medicine, Atlanta, USA; 3 School of Medicine and Public Health, Department of Medicine, University of Wisconsin, Madison, USA; 4 School of Medicine and Public Health, Department of Medicine, Division of Nephrology, University of Wisconsin, Madison, USA

**Keywords:** Biopsy, Kidney Biopsy, Complications, Risk, Bleeding

## Abstract

**Background:**

Percutaneous kidney biopsies are important tools for the diagnosis of kidney diseases. Nephrologists must be familiar with the expected complications of the procedure to provide an adequate informed consent. Here, we present a quality improvement analysis that reviews the complication rate of percutaneous kidney biopsies performed over a 2-year period by nephrologists at a single center, and that tabulates the nature and timing of these events.

**Methods:**

From a single center cohort, pre- and post-biopsy anthropomorphic and clinical measurements were collected. Post-biopsy complications were tracked and sorted into either major or minor complications. Statistical tests were used to analyze complication incidence across the pre- and post-biopsy measurements obtained.

**Results:**

Of the 154 nephrologist-performed percutaneous native kidney biopsies, 2 biopsies (1.3%) were found to result in a major complication. Both major complications were detected within 4 hours of the biopsy. Analysis of the pre-biopsy and post-biopsy measurements found that the proportion of complications was higher in patients with hematuria prior to biopsy. It was also found that patients with complications were statistically younger and had fewer comorbidities. Under univariable analysis, older age was associated with a lower incidence rate ratio for complications. However, no pre-or-post biopsy measurement or characteristic had a statistically significant change in incidence rate ratio under multivariable analysis.

**Conclusions:**

Percutaneous kidney biopsies were found to be low risk when performed by nephrologists in this single center cohort. Consistent with past literature, life threatening major complications rarely occurred and were reliably identified within 4 hours of biopsy, suggesting that centers can consider reduced observation times without compromising patient safety. Minor complications, such as pain, were more likely to occur in younger, healthier patients, and in those with hematuria prior to biopsy. This extensive tabulation of all biopsy adverse events is the first of its kind and will be beneficial for nephrologists to inform discussions with patients about expectations and risk-benefit of this procedure.

## Introduction

Percutaneous kidney biopsies are considered the gold standard for diagnosis and management of kidney diseases [1]. They have historically been performed by nephrologists, however, in the recent years, radiologist performed percutaneous kidney biopsies have become more common [2]. Nevertheless, kidney biopsies remain an essential part of nephrology practice. This is evidenced by requirements from the Accreditation Council on Graduate Medical Education and the American Board of Internal Medicine for competence in percutaneous biopsies of both native and allograft kidneys for all nephrology fellows [1]. This education remains important because even in biopsies performed by radiologists, it is likely that the nephrologist ordered the procedure and discussed the risks and benefits with the patient. Thus, it is important for nephrologists to remain familiar with the safety profile and post-operative events related to kidney biopsies.

The significant vascularity and the large amount of cardiac output the kidney receives relative to its size, lends the procedure to bleeding complications. Several large retrospective and prospective studies exploring biopsy complications have shown that bleeding related complications are most common and can be quite frequent (50–90%) [1, 3–6]. However, these often-cited numbers are based on older 1980s studies. Major bleeds resulting in emergency intervention such as transfusion or embolization are uncommon but had a wide range of incidence (< 1 to 9%) [3, 7–9]. Timing of complications is also variable, and this has led to controversy regarding the optimal length of post-procedure monitoring. Some studies advocate for observation periods up to 24 hours while others suggest that shorter observation times are sufficient to catch major complications [8, 10–12]. Many of these published reports include biopsies performed by other specialists such as urologists and radiologists. The variability of biopsy indication may account for the wide range of complications found in the literature.

The aim of this quality improvement analysis was to contribute to the existing data on the safety of percutaneous kidney biopsy with a specific focus on nephrologists performing the procedure for nephrology specific indications. It was anticipated that our findings would match existing data for biopsy safety and be able to provide reassurance that biopsies performed by trained nephrologists are low risk. The standard post-procedure observation period at our center was also assessed to determine if it could be safely decreased.

## Methods

### Study patients

A retrospective chart review was performed on patients who received an outpatient referral for native kidney biopsy at the University of Wisconsin Hospitals and Clinics between January 2016 and January 2018. Any biopsy done outside the nephrology division was excluded from the study. Per typical protocol, patients were asked to hold antiplatelet therapy and anticoagulants for 2–5 days prior to the biopsy (2 days for direct oral anticoagulants (DOACs) and 5 days for aspirin, clopidogrel and warfarin). The patients were admitted to the hospital prior to the biopsy and had baseline blood work drawn (hemoglobin, hematocrit, platelet count, INR). The procedure occurred in the radiology suite with an ultrasound technologist guiding a nephrologist using an 18-gauge side cutting percutaneous biopsy device for the majority of procedures. There were a few procedures done with 14- and 16-gauge end cutting needles, but this frequency was not tracked. A nephrology attending physician was present for each biopsy where a nephrology fellow was the primary operator. After the procedure, the patient was returned to their inpatient room for observation. Nursing staff were instructed to measure vital signs every 15 minutes for 1 hour then every 30 minutes for 1 hour and then every 4 hours thereafter. Pain was assessed at each vital sign observation using the visual analogue scale. Blood work was drawn at 4 hours and again in the morning. If the hematocrit levels were stable, the patient was discharged home with instructions to return to the hospital for any significant increase in pain, lightheadedness, dizziness or other change in condition. If the hematocrit fell by more than 3 points at either check, a non-contrast CT scan was ordered to assess for bleeding.

### Study variables

Two medical student research assistants defined study variables in consultation with the study principal investigators and developed a password-protected electronic abstraction form in Microsoft Office Excel. A codebook was developed for each categorical variable with comprehensive, mutually exclusive numerical codes to ensure accuracy and consistency. Variables included major complications (need for transfusion, embolization, death) and minor complications (pain, nausea, drop in hematocrit > 3 points, stable hematomas, low grade fever (subjective fever and/or recorded temperature > 99.6 °F and < 100.4 °F), hypertension (> 180/110 mmHg), and hypotension (< 90/60 mmHg)).

Patients were randomly assigned to a research assistant’s panel for data abstraction. The frequency, timing, and nature of kidney biopsy related complications was recorded. Patient identifying factors were eliminated in accordance with quality improvement principles. Pre and post biopsy characteristics were analyzed for predictive potential for complications. The first 10 patients in each research assistant’s data set were assigned twice and re-coded to ensure inter-rater and intra-rater reliability. No coding discrepancies were identified.

### Statistical analysis

To determine if any patient characteristics were associated with kidney biopsy complications, we performed the following analyses. Baseline characteristics and patient demographic information were summarized using proportions and Fisher’s exact tests. Continuous variables such as age, BMI and Charlson score were expressed as mean plus standard deviation and analyzed using t-tests. We modelled the incidence of complications using Kaplan Meier Incidence curves and a Poisson regression. Both a univariable and a multivariable Poisson model were conducted with variables deemed risk factors for complications. Our multivariable model was conducted using all variables included in the univariable model, with exclusions only made in cases of redundancy. Missing data points were handled by deletion/omission from modelling in listwise fashion. All *p*-values < 0.05 were considered statistically significant. All analyses were conducted using STATA version 15 SE (StataCorp. 2017. *Stata Statistical Software: Release 15. College Station, TX: StataCorp LLC*).

## Results

Of the 178 patients sent for percutaneous biopsy, 154 were included in the study (Table 1). Twenty-four patients were excluded because they either received a biopsy outside the nephrology division or at an alternative institution. All the included 154 patients underwent native kidney biopsy, performed by 20 nephrologist attendings and fellows. The sample was 51.9% men, with an average age of 54.9 years and an average BMI of 30.2Kg/m^2^. Patient comorbidity was estimated with the Charlson Comorbidity Index, of which a higher index predicts a lower 10-year survival. The average Charlson Comorbidity Index of our cohort was 3.69, which estimates a 62% 10-year survival. The diagnoses resulting from biopsy are also shown. All 154 biopsies resulted in sufficient tissue for pathology reading. Under the category of “Others,” we included low volume diagnoses (occurring less than three time) such as amyloidosis, HIV nephropathy, light chain tubulopathy, pyelonephritis, radiation induced kidney injury, thin basement membrane disease, thrombotic microangiopathy and microangiopathy likely due to bone marrow transplant. While interesting to note, these did not result in large enough numbers to be associated with any biopsy complication therefore were not listed individually. Pathology readings of non-specific renal changes occurred 9 times and were classified as “other” due to not being a renal disease diagnosis.

**Table 1:** Biopsy patient characteristics

Total Biopsies	154
**Characteristics**	
Male %	51.9
Age, years, mean (SD)	54.9 (16.6)
Body Mass Index in kg/m^2^, mean (SD)	30.2 (8.5)
Charlson Comorbidity Index, mean (SD)	3.69 (2.48)
**Biopsy Results**	
Diabetic Nephropathy, N (%)	35 (22.7)
Interstitial Nephritis, N (%)	16 (10.4)
Focal Segmental Glomerulosclerosis, N (%)	16 (10.4)
Lupus Nephritis, N (%)	14 (9.1)
Nephrosclerosis due to Hypertension	12 (7.8)
IgA Nephropathy, N (%)	12 (7.8)
Membranous Nephropathy, N (%)	7 (4.5)
Membranoproliferative Glomerulonephritis, N (%)	5 (3.3)
Medication-Induced Renal Complications, N (%)	5 (3.3)
Immune Complex Glomerulonephritis, no further comment, N (%)	4 (2.6)
ANCA Vasculitis, N (%)	3 (1.9)
Others N (%)	25 (16.2)

Abbreviations: *kg/m*^*2*^ kilograms per meter squared, *SD* Standard Deviation. Diagnoses under “Others” include amyloidosis, HIV nephropathy, light chain tubulopathy, pyelonephritis, radiation induced kidney injury, thin basement membrane disease, thrombotic microangiopathy, microangiopathy likely due to bone marrow transplant, and non-specific renal changes. “Medication-Induced Renal Complications” were distinguished from “Interstitial Nephritis” in that a specific medication was a recognized as a cause for renal injury at the time of diagnosis. Medication-Induced Renal Complications included PPI induced allergic interstitial nephritis, NSAID induced CKD, and chemotherapy induced CKD

Among our five biopsies that resulted in MPGN, three were thought to be due to immune complex mediated disease, one was thought to be due to cryoglobulinemia, and one was not further classified. Major complications.

Two biopsies (1.3%) resulted in a major complication (Table 2). One biopsy resulted in gross hemorrhage noted as an ongoing hemorrhage on CT scan. Embolization was planned for this patient, but bleeding stabilized, and subsequent hematocrits remained stable. The other major complication required a transfusion after the hemoglobin had dropped below 7 g/dL. Further imaging showed bleeding had stopped therefore no further intervention was necessary. Both of the 2 major complications, gross hemorrhage and need for transfusion, were identified within 4 hours of biopsy.

**Table 2:** Frequency and timing of major and minor complications post-kidney biopsy

	Total Complications ***N*** = 108	Major Complications ***N*** = 2	Minor Complications ***N*** = 106
**Time Frame Post Biopsy**	# of Complications (%)	Complication Description (# of times occurred)	Complication Description (# of times occurred)
**0–4 hours**	54 (50)	Gross Hemorrhage (1) Transfusion Needed (1)	Pain (28) Drop in Hematocrit (7) Stable Hematoma (11) Nausea/Vomiting (3) Hypotension (2) Low Grade Fever (1)
**4–8 hours**	38 (35.2)		Pain (19) Drop in Hematocrit (17) Hematoma (1) Hypertension (1)
**8–12 hours**	7 (6.5)		Pain (3) Drop in Hematocrit (4)
**12h hours**	9 (8.3)		Pain (3) Drop in Hematocrit (2) Stable Hematoma (1) Hypertension (1) Hypotension (1) Fever (1)

Of note, the same patient may have reported more than one symptom, and each would be reported as a different instance.

### Minor complications

The remaining complications were considered minor complications. The most common minor complication reported was pain; reported in 53 biopsies. The average subjective rating on the pain visual analogue scale was 3.1/10 at rest and 4.0/10 with activity. The next most common minor complication was a decrease in hematocrit, occurring in 30 biopsies. A drop of 3 or more points in hematocrit was followed with abdominal CT scan 29 times to search for hemorrhage, which occurred once. All other CT scans or ultrasounds revealed small hematomas, occurring 13 times, or showed expected post kidney biopsy changes such as trace peri-nephric blood.

The total number of minor complications documented 106. Most complications, 54(50%), were reported within 4 hours after biopsy, followed by 38 (35.2%) complications reported within 4–8 hours post biopsy, followed by 7 (6.5%) reported within 8–12 hours post biopsy, and the remaining 9 (8.3%) of complications reported 12 hours post biopsy. (Table 2).

### Characteristics contributing to complications

Table 3 summarizes the differences between patients who experienced any major or minor complication and those who did not experience any complication. While not significant, minor complications occurred in patients with hematuria prior to biopsy more often than those without. The most common minor complication in patients with hematuria pre-biopsy was pain, occurring in 30/41 (73%) of patients with hematuria pre-biopsy, followed by a decrease in hematocrit > 3 points, occurring in 14/41 patients (34%). Complication risk was the same between men and women. There was no difference in complication occurrence between those taking ACE/ARBs, antiplatelet agents, or other anticoagulants and those who did not. Antiplatelet agents and anticoagulants were held for 2–5 days prior to the biopsy. For biopsies performed on those who had proteinuria, the proportion of complications was not significantly different from those who did not have proteinuria.

**Table 3:** Comparison of pre-biopsy variables

	Total ***N*** = 154 Pts	No Complications ***N*** = 79 Pts	Complications ***N*** = 75 Pts	*P*-Value
**Male, N (%)**	80 (51.9)	43 (54.4)	37 (49.3)	0.63
**ACEi/ARB Use, N (%)**	67 (43.5)	35 (44.3)	32 (42.7)	0.87
**Anticoagulant Users, N (%)**	14 (9.0)	6 (7.6)	8 (10.7)	0.58
**Anti-Platelet Users, N (%)**	37 (24.2)	21 (26.9)	16 (21.3)	0.45
**Proteinuria Pre Bx, N (%)**	124 (80.5)	61 (77.2)	63 (84.0)	0.32
**Hematuria Pre Bx, N (%)**	71 (46.1)	30 (37.9)	41 (54.7)	0.05*
**Hypertension Pre Bx, N (%)**	77 (50.0)	39 (49.4)	38 (50.6)	1.00
**Age years, mean (SD)**	54.9 (16.6)	58.9 (17.1)	50.7 (15.1)	0.002*
**Body Mass Index, kg/m**^**2**^**, mean (SD)**	30.2 (8.5)	29.7 (8.46)	30.8 (8.53)	0.39
**Charlson Score, mean (SD)**	3.69 (2.48)	4.2 (2.64)	3.16 (2.2)	0.008*
**Systolic Blood Pressure, mmHg, mean (SD), data not available for 3 pts**	136.0 (17.8)	135.7 (18.1)	136.3 (17.5)	0.85
**Diastolic Blood Pressure, mmHg, mean (SD), data not available for 3 pts**	80.47 (10.9)	80.3 (9.68)	80.6 (12.1)	0.85
**Platelet Count Pre-Biopsy, /uL, mean (SD)**	251.7 (77.0)	255.0 (72.1)	248.2 (82.1)	0.58
**INR Pre-Biopsy, mean (SD)**	1.0 (0.13)	1.0 (0.09)	1.0 (0.16)	0.52
**Hemoglobin Before-Biopsy, g/dL, mean (SD)**	12.1 (2.2)	12.1 (2.1)	12.1 (2.2)	0.98
**Hemoglobin After-Biopsy, g/dL, mean (SD), data not available for 5 pts**	11.6 (2.2)	11.7 (2.2)	11.5 (2.2)	0.57

Abbreviations: */uL* per microliter, *ACEi* Angiotensin Converting Enzyme Inhibitor, *ARB* Angiotensin Receptor Blocker, *Bx* biopsy, *g/dL* grams per deciliter, *INR* International Normalized Ratio, *kg/m*^*2*^ kilograms per meter squared, *mmHg* millimeters of mercury, *Pts* Patients, *SD* Standard Deviation

Among age it was found that the average age of patients who underwent biopsies that resulted in minor complications was significantly lower than those who did not have complications (Table 4). Similarly, the average Charlson comorbidity index was significantly lower among those who had minor complications. Under univariable analysis, age was the only variable that demonstrated a significant change in incidence. Under multivariable analysis, none of the variables demonstrated a significant change in the incidence rate ratio.

**Table 4:** Analysis of pre-biopsy variables

	Univariable			Multivariable		
	IRR	95 CI	P-Value	IRR	95 CI	*P*-Value
**Age**	0.98	0.97–0.99	0.03*	0.99	0.98–1.02	0.92
**Elevated Blood Pressure (> 140/90 mmHg)**	1.32	0.73–2.4	0.35	1.08	0.55–2.10	0.82
**Gender Male**	0.90	0.57–1.41	0.65	1.06	0.63–1.78	0.82
**Body Mass Index**	1.01	0.98–1.03	0.54	1.00	0.97–1.03	0.95
**Charlson Score**	0.65	0.41–1.02	0.06	1.05	0.54–2.04	0.87
**Systolic Blood Pressure, data not available in 3 patients**	1.0	0.99–1.01	0.89	–	–	–
**Diastolic Blood Pressure, data not available in 3 patients**	1.0	0.98–1.02	0.89	–	–	–
**Platelet Count Pre-Biopsy**	0.99	0.99–1.00	0.69	0.99	0.99–1.00	0.89
**INR Pre-Biopsy**	0.68	0.14–3.37	0.64	1.12	0.24–5.27	0.87
**ACE/ARBS Use**	0.97	0.61–1.52	0.88	1.07	0.63–1.84	0.79
**Anticoagulant Use**	1.19	0.57–2.48	0.63	0.99	0.42–2.35	0.99
**Anti-platelet Use**	0.85	0.49–1.48	0.56	–	–	–
**Proteinuria**	1.27	0.68–2.35	0.45	1.09	0.57–2.11	0.78
**Hematuria**	0.98	0.55–1.75	0.94	1.03	0.54–1.94	0.93

Abbreviations: *95 CI* 95% confidence interval, *ACEi* Angiotensin Converting Enzyme Inhibitor, *ARB* Angiotensin Receptor Blocker, *INR* International Normalized Ratio, *IRR* Incidence Rate Ratio

The small number of major complications (*n* = 2) limited statistical analysis that could be performed for this group.

## Discussion

This quality improvement study analyzed t 154 patients who underwent native percutaneous kidney biopsies performed by a nephrologist, to determine complication rate at our center, and to determine patient characteristics associated with kidney biopsy complications . There was a major complication rate of 1.3%, which is in line with the reported range of < 1–9% [4–7]. For comparison, a recent retrospective study on appendectomies reported major complications occurring in roughly 1.8% of procedures [13]. This aforementioned study applied the Clavien-Dindo classification, which grades complications based on the degree of invasiveness required for correction. The interventions required for appendectomy complications (grades III-V) were much more invasive than those required after kidney biopsies (grade I and II). In other words, the appendectomy, a procedure that is considered common and low risk, has major complications that occur more often and require more invasive interventions according to our data. This comparison can help inform patients of their risk.

In our population, minor complications were found in 47% of biopsies. All these complications resolved with minimal to no intervention within 24 hours of the procedure. Table 2 provides the frequency of issues such as pain and nausea surrounding the biopsy. This is the first time this type of information exists in the literature. While these issues are not clinically significant, understanding the frequency of occurrence can allow for both improved patient expectations and stronger patient education. In surgical literature, patient education on what to expect can facilitate recovery, and minimizes pain, anxiety, and functional issues [ 14–16].

It was noted that minor bleeding complications resulting in a hematoma were reported in only 13 biopsies or 8.4%. This contrasts with many reported values ranging from 57 to 91% [1]. However, in this study, hematomas were detected after an indication for imaging (declining hematocrit, persistent flank pain, etc.), and thus do not represent asymptomatic hematomas or those that occurred in patients with stable hematocrits. This is the likely explanation for the discrepancy.

Although none of the collected data stood out in multivariate analysis, it was interesting to note that younger patients and those with lower Charlson comorbidity indices were more likely to report minor complications. Younger age has been reported in the literature as a risk factor for kidney biopsy complications [17, 18] but minor complications have not previously been associated with low comorbidities. Pain represented about half of the minor complications. Patients who are younger with fewer comorbidities likely have less conditioning to uncomfortable medical situations and therefore, the subjective pain threshold for them may be lower than in older patients with more comorbidities. In conversations with younger, healthier patients, nephrologists can use this information to discuss expectations regarding pain and nausea, as they occur with regularity, and other minor complications tabulated here.

Although the literature has shown there to be an increased bleeding risk associated with diagnoses such as acute tubular necrosis, autoimmune conditions, and hypertensive kidney disease, no associations have been substantiated consistently [19]. Given the low rate of major complications, our cohort did not support any association. There was an increased incidence of minor complications associated with hematuria, but the reason is unclear. Nevertheless, the tabulation of biopsy diagnoses provided in Table 1 may be of use to other centers, in that they could expect a different incidence of complications if their patient population has a different make-up of kidney diseases.

Periods of observation after a kidney biopsy have come into question. Although collection methods differ, past literature supports that 85–91.6% of complications occurred 12 hours or less from the time of the biopsy [1, 9]. More contemporary studies suggested that 100% of major complications were reported in 5 hours of the kidney biopsy [10]. Indeed, as a result of our findings, our center transitioned in mid-2019 from a 24-hour observation period to an observation period of 4 hours with one blood draw at the end of observation. To date, no unexpected issues regarding post-biopsy complications or hospital readmissions have been observed.

Studies have also suggested that the risk of complications in kidney biopsies is reduced when the performer has done greater than 4 biopsies per year [20]. While the data we provided suggest our nephrologists average roughly 3.8 biopsies per year, it should be noted that this study investigated complication rates in patients who received only outpatient referrals for kidney biopsy. A considerable portion of the biopsies performed by this same group of nephrologists also comes from inpatient native renal biopsies which would raise the number of biopsies well above the 4/year threshold. Consideration for the number of procedures per year per nephrologist should be taken when creating a system that places patient safety at the forefront.

Our study provides information on the low rate of major complications in a single center where nephrologists perform the kidney biopsies for nephrology indications. In addition to this contemporary update, we provide information on the minor complications to inform nephrologists’ discussions about what patients can expect. In addition, we provide evidence that supports prior reports that complications most often exist in the 4–5 hours post biopsy, therefore observation periods can reflect this practice safely. There were limitations in our study given its single center, observational nature and relatively small sample size as this restricted the number of major complications and limited the statistical analysis that could be performed. Additionally, we did not track the type of biopsy device that was used, nor the number of passes taken. These factors have been shown to influence the complication rate [21]. Finally, the retrospective nature of our data collection also subjected our study to the disadvantages that are inherent to retrospective studies, such as the inability to control assessment of outcomes and susceptibility to unmeasured confounding factors. Together, these limitations could limit the generalizability of our findings.

## Conclusion

Our study was successful in providing further evidence to support the safety of percutaneous kidney biopsies, with major complication rates comparable to the rates in existing literature. The strengths of our study, however, were found in the extensive tabulation of events. Through the tabulation of timing, we were able to show that shorter observation periods could limit hospital stays without compromising patient safety. Through the tabulation of post-biopsy complications, we added to the existing knowledge of post-biopsy expectations. These tabulations also allowed the statistical analysis which showed minor complications to be more frequent in younger, healthier patients. Despite the limitations of being a small, retrospective, single center study, we believe our study is a good starting point towards obtaining aclearer picture of the incidence of events such as pain and nausea, that will allow nephrologists tomore confidently and adequately educate their patients about what to expect when undergoing a kidney biopsy. To expand on our findings, future studies can examine the effect of different gauge needles, the number of passes in a biopsy, and the experience of the biopsy performer on the post-biopsy complication rate.

## Data Availability

The datasets used and/or analysed during the current study are available from the corresponding author on reasonable request.
